# Save lives and relieve suffering: The twin imperatives of humanitarian response and the role of palliative care

**DOI:** 10.3389/fonc.2023.1120380

**Published:** 2023-03-02

**Authors:** Bethany-Rose Daubman, Farzana Khan, Sarah E. Slater, Eric L. Krakauer

**Affiliations:** ^1^ Division of Palliative Care and Geriatric Medicine, Department of Medicine, Massachusetts General Hospital and Harvard Medical School, Boston, MA, United States; ^2^ Fasiuddin Khan Research Foundation, Palliative Care Division, Dhaka, Bangladesh; ^3^ University of Edinburgh, Global Health Division, Edinburgh, United Kingdom; ^4^ Department of Medical Oncology, Division of Gastrointestinal Oncology, Dana Farber Cancer Institute and Harvard Medical School, Boston, MA, United States; ^5^ Department of Palliative Care, University of Medicine & Pharmacy at Ho Chi Minh City, Ho Chi Minh City, Vietnam

**Keywords:** palliative care, cancer, humanitarian response, humanitarian emergencies and crises, oncology

## Introduction

Humanitarian emergencies and crises (HECs) are large-scale events that often cause injuries, illness or death on a massive scale, social breakdown, forced displacement, and all types of physical, psychological, social, and spiritual suffering. They rarely are caused by a single factor and are usually the result of mixed climatic/geologic, human-made, environmental, political and economic causes and vulnerabilities (1, 2). The principles of humanitarianism explicitly require efforts both to save lives and to prevent and alleviate human suffering, and these two imperatives rarely are in conflict (1, 3–5). Yet throughout the world, humanitarian medical response focuses primarily on saving lives and lacks adequate attention to preventing and relieving suffering (6–9). This moral failing appears to have several causes including lack of adequate palliative care content in humanitarian response guides and training; legal or logistical barriers to opioid access in fragile, conflict-affected, and vulnerable settings; and negative attitudes toward palliative care (10).

## Palliative care in humanitarian emergencies and crises

Palliative care entails prevention and relief of serious physical, psychological, social or spiritual suffering of patients, chronic or acute, and psychological, social and spiritual suffering of their family members (1). The World Health Organization (WHO) recognizes that the specific types, scale, and severity of suffering often varies by geopolitical location, by economic situation, by culture, and, in the setting of HECs, by the type of emergency or crisis (1). But basic palliative care training and supplies are adequate to alleviate most types of suffering in any situation. In HECS, services to prevent and relieve suffering should be made accessible for anyone suffering physically, psychologically, socially, or spiritually and not only for those with life-threatening conditions. There is a particular ethical imperative, based on the medical and ethical principles of beneficence and non-abandonment, to providing palliative care and symptom control for patients whose death is deemed unavoidable under prevailing circumstances. Failure to benefit these highly vulnerable patients by treating aggressively and immediately any physical and psychological suffering constitutes abandonment and is thus ethically unacceptable. Yet it is crucial to avoid equating palliative care with end-of-life care and instead to recognize that palliative care should be provided in response to any moderate or severe suffering in an HEC, regardless of the patient’s prognosis (1, 11).

## Palliative cancer care in humanitarian emergencies and crises

As Caglevic et al. powerfully state, “war breeds cancer—delaying diagnosis, preventing treatment, and increasing risk (12).” Hospitals often redirect care to trauma victims, leaving fewer resources available to manage medically fragile patients with cancer or other serious illness (13). HECs, especially armed conflict, disrupt and prevent cancer treatment, divert resources from oncologic care, expose vulnerable patients with cancer to additional risks such as infection, and lead to delays in diagnosis (12). Delaying surgery for breast cancer by 12 weeks leads to a 26% increase in mortality, with similar findings reported for bladder, colon, head and neck, and non–small-cell lung cancers (14). All of these factors lead to an increased need for palliative care in patients with cancer in HECs.

Palliative care is never an acceptable alternative to cancer prevention, diagnosis, and treatment. Efforts always should be made to assure access to comprehensive cancer care including prevention, early diagnosis, and treatment. But while access to cancer chemotherapy or radiotherapy may be lost in an HEC, access to palliative care always can and should be maintained. Using the essential package of palliative care for humanitarian emergencies and crises (EPPCHECs) recommended by WHO, palliative care is relatively simple to provide in areas where more comprehensive cancer care may not be immediately available, such as areas of conflict (1).

While it is never ethically permissible to regard palliative care as a substitute for comprehensive cancer care, the reverse is also true; it is not ethically permissible to focus on cancer prevention and treatment while ignoring the responsibility to palliate the suffering of patients and families (15). It has been shown that palliative care can prolong the lives of some cancer patients (16). Additionally, chemotherapy, radiation, and surgery are all aspects of comprehensive palliative care for patients whose cancer is incurable and can be a valuable means of addressing suffering. Thus, there is a not a dichotomy between cancer treatment and palliative care.

Lastly, as palliative care emphasizes attention to social and family histories, personal values, and whole-person care, the inclusion of palliative care in response to humanitarian crises can also promote cultural sensitivity and adaptation to local culture. It is vital that any humanitarian response be attuned to local culture and include local people. A key principle of palliative care integration in HECs is recognition of local colleagues’ expertise, with a focus on assisting local colleagues to do the work needed and to build or rebuild healthcare infrastructure. Part of this role would be providing training in palliative care and assistance in developing culturally-adapted, sustainable palliative care services (and other cancer services).

## Discussion

To make palliative care accessible to all in need in HECs, it should be thoroughly integrated into international and national humanitarian response protocols and organizations. This will require significant but feasible and affordable changes in humanitarian response policy, clinical guidance, triage, training, essential supplies, and indicators. The leading humanitarian response organizations, including the United Nations High Commission on Refugees (UNHCR), the International Committee of the Red Cross (ICRC), the International Federation of Red Cross and Red Crescent Societies (IFRC), the Doctors Without Borders organizations (MSF), the Save The Children organizations, Catholic Relief Services (CRS), and others, should make clear in their policy statements and field guides that prevention and relief of physical, psychological, social, and spiritual suffering with palliative care is “an ethical responsibility” (17). We applaud the editors of the 2018 revision of The Sphere Handbook, the world’s leading handbook of humanitarian response, for inviting one of us (ELK) to contribute a section on palliative care as one of the “minimum standards in humanitarian response” (4). However, the final edited version propagates the misconception that palliative care is only for dying patients. Future guidance documents should emphasize the twin imperatives of saving lives and relieving suffering and make clear that palliative care is not only for the dying.

In developing policies and clinical guides, it is crucial that conflict over nomenclature or “turf” be avoided between experts in related fields such as anesthesia, mental health, social support, and palliative care. Excellent guidance on mental health and psychosocial support in emergency situations already exists, and social supports already are a standard part of most humanitarian response (18–20). Whether psychological first aid, for example, is called mental health care or palliative care, and whether relief of acute pain from traumatic injury is called anesthesia or palliative care, is unimportant. What matters is that humanitarian responders be trained to provide all needed services and that guidance on saving lives, on preventing and relieving physical, psychological, social, and spiritual suffering, and on strengthening local health care systems be easily accessible in one place and in many languages. Humanitarian responders cannot be expected to access multiple manuals on all relevant topics.

Palliative care should be an integral part of triage protocols. Allocation of “expectant” patients, those whose survival is not possible with available resources, to the color black at the bottom of triage protocols suggests that they be abandoned and is unethical. We support the WHO proposal of a revised triage protocol that includes palliative care at all levels and makes clear that expectant patients require care (**Table 1**).

**Table 1:** WHO recommended triage categories in humanitarian emergencies and crises (1).

Category	Colour code	Description
1a. Immediate	Red	Survival possible with immediate treatment
		Palliative care should be integrated with life-sustaining treatment as much as possible.
2a. Expectant	Blue	Survival not possible given the care that is available
		Palliative care is require
	Yellow	Not in immediate danger of death, but treatment needed soon.
		Palliative care and/or symptom relief may nevertheless be needed immediately.
3. Minimal	Green	Will need medical care at some point, after patients with more critical conditions have been treated.
		Symptom relief may be needed.

The colors are adapted by the World Health Organization from colors of standard triage categorization in humanitarian emergencies and crises.

The WHO essential package of palliative care for humanitarian emergencies and crises (EPPCHECs) should be a standard part of the equipment of all international and national humanitarian response organizations. The EPPCHECs includes a set of safe, effective, inexpensive, off-patent and widely available medicines, simple and inexpensive equipment, basic social supports, and the trained human resources needed to apply them appropriately, effectively, and safely (1). Morphine, in oral fast-acting and injectable preparations, is the most clinically important of the essential palliative care medicines and should be accessible in all HECS to treat moderate or severe pain and refractory terminal dyspnea (21). Although WHO has guidelines designed to enable emergency import of controlled medicines for HECs (22), humanitarian responders often report that morphine and other controlled medicines are not available or that efforts to import them for humanitarian response often prove futile (23, 24). The need for access to basic palliative care medications, especially pain medications, is especially great in cancer patients whose care is likely to be disrupted in HECs. Thus, the mechanism for emergency importation of controlled medicines requires revision based on global consensus. Maximizing accessibility of controlled medicines for medical uses must be balanced by reasonable precautions to prevent diversion and non-medical use. Model guidelines for this purpose are available (25).

Additionally, training is an integral component of implementing palliative care in HECs, especially for patients with cancer. Humanitarian responders should be trained in palliative care (basic or intermediate training—not just for cancer patients, but for many patients for whom palliative care skills are needed).

Finally, a standard instrument for data collection on humanitarian response is needed to assess not only patients’ diagnoses and mortality rates but adequacy of palliative care such as those developed by WHO and The Sphere Handbook (4, 26, 27).

## Conclusion

People with cancer and other serious illnesses are at particular risk of suffering in the settings of HECs. Access to palliative care is crucial to realization of the human right to the highest attainable standard of health and well-being, and it is an essential component of comprehensive care for people with these illnesses. Under normal circumstances, it should be integrated with prevention, diagnosis, and treatment of such illnesses and should never be considered an acceptable alternative to them. But when such services are disrupted or unavailable due to conflict or other crises, both the need for palliative care and the moral imperative to provide it increase dramatically. Provision of the WHO essential package of palliative care remains feasible even when other services such as chemotherapy, radiation therapy, and surgery are not. To ensure access to palliative care for people affected by humanitarian crises, humanitarian response policies and guidance documents should include palliative care, all humanitarian responders should be trained and equipped to provide palliative care, and research on humanitarian crises should include indicators of palliative care need, provision, and quality.

## Author contributions

All authors contributed to the writing and editing of this article and approved the submitted version.
